# Supplementary material for: The Systems Biology Research Tool: evolvable open-source software
Source: BMC Syst Biol. 2008 Jun 29;2:55. doi: 10.1186/1752-0509-2-55 (PMC2446383; doi:10.1186/1752-0509-2-55)
Supplement: Additional file 1 — SBRT Archive. An archive of the current version of the Systems Biology Research Tool. [file 1752-0509-2-55-S1.zip › sbrt-1.4.0/doc/developers_guide/api/sbrt/kernel/math/AbstractPolynomial.html]

AbstractPolynomial


|  |  |  |
| --- | --- | --- |
| **Overview** \| **Package** \| **Class** \| **Use** \| **Tree** \| **Deprecated** \| **Index** \| **Help** | |  |
| **PREV CLASS**   **NEXT CLASS** | **FRAMES**    **NO FRAMES**     **All Classes** |  |
| SUMMARY: NESTED \| FIELD \| CONSTR \| METHOD | DETAIL: FIELD \| CONSTR \| METHOD |  |


---


## sbrt.kernel.math Class AbstractPolynomial<V>

```
java.lang.Object
  sbrt.kernel.math.AbstractPolynomial<V>
```

**Type Parameters:**: `V` - the variable type.

**All Implemented Interfaces:**: MathExpr<V>, Polynomial<V>

**Direct Known Subclasses:**: AbstractLinearComb

---

``` public abstract class AbstractPolynomial<V> extends java.lang.Object implements Polynomial<V> ```

This abstract class is a skeleton implementation of `Polynomial`.

**Author:**
:   This class was written and documented by Jeremiah Wright
    while in the Wagner lab.

---

**Nested Class Summary**

**Nested classes/interfaces inherited from interface sbrt.kernel.math.Polynomial**

`Polynomial.Term<V>`


**Constructor Summary**

`AbstractPolynomial(double constant)`
  
          Constructs a new polynomial with the provided constant.


| **Method Summary** | |
| --- | --- |
| `protected static void` | `checkConstant(double constant)`             Ensures the provided constant is not infinite and not NaN. |
| `double` | `getConstant()`             Returns the constant of this polynomial. |
| `int` | `hashCode()`             Returns a content-based hash code of this polynomial. |
| `java.lang.String` | `toString()`             Returns a string representation of this polynomial. |

**Methods inherited from class java.lang.Object**

`clone, equals, finalize, getClass, notify, notifyAll, wait, wait, wait`

**Methods inherited from interface sbrt.kernel.math.Polynomial**

`equals, getTermArray, plugInNumbers, terms`

**Methods inherited from interface sbrt.kernel.math.MathExpr**

`getVariables, hasAllVariables, hasVariable, hasVariables, variables`

**Constructor Detail**

### AbstractPolynomial

```
public AbstractPolynomial(double constant)
```

:   Constructs a new polynomial with the provided constant.

    **Parameters:**: `constant` - the constant of this polynomial. **Throws:**: `java.lang.IllegalArgumentException` - if the provided constant is either infinite or NaN.


**Method Detail**

### getConstant

```
public double getConstant()
```

:   Returns the constant of this polynomial.

    :   **Specified by:**: `getConstant` in interface `MathExpr<V>` **Specified by:**: `getConstant` in interface `Polynomial<V>`
    :   **Returns:**: the constant of this polynomial.

---


### hashCode

```
public int hashCode()
```

:   Returns a content-based hash code of this polynomial.

    :   **Specified by:**: `hashCode` in interface `MathExpr<V>` **Specified by:**: `hashCode` in interface `Polynomial<V>` **Overrides:**: `hashCode` in class `java.lang.Object`
    :   **Returns:**: a content-based hash code of this polynomial.

---


### toString

```
public java.lang.String toString()
```

:   Returns a string representation of this polynomial.

    :   **Specified by:**: `toString` in interface `MathExpr<V>` **Specified by:**: `toString` in interface `Polynomial<V>` **Overrides:**: `toString` in class `java.lang.Object`
    :   **Returns:**: a string representation of this polynomial.

---


### checkConstant

```
protected static void checkConstant(double constant)
```

:   Ensures the provided constant is not infinite and not NaN.

    :   **Parameters:**: `constant` - the constant to check. **Throws:**: `java.lang.IllegalArgumentException` - if the provided constant is either infinite or NaN.


---


|  |  |  |
| --- | --- | --- |
| **Overview** \| **Package** \| **Class** \| **Use** \| **Tree** \| **Deprecated** \| **Index** \| **Help** | |  |
| **PREV CLASS**   **NEXT CLASS** | **FRAMES**    **NO FRAMES**     **All Classes** |  |
| SUMMARY: NESTED \| FIELD \| CONSTR \| METHOD | DETAIL: FIELD \| CONSTR \| METHOD |  |


---
